# Place of death in France: impact of the first wave (March–May 2020) of the Covid-19 epidemic

**DOI:** 10.1186/s12889-023-15651-6

**Published:** 2023-04-28

**Authors:** Néstor Aldea-Ramos, Yann Le Strat, Anne Fouillet

**Affiliations:** Division of Data Science, Santé Publique France, 12 Rue du Val d’Osne, Saint-Maurice, 94410 France

**Keywords:** Place of death, COVID-19, Mortality surveillance, France

## Abstract

**Background:**

The Covid-19 epidemic entailed a major public health issue in France challenging the efficiency of the public health system. The distribution of deaths by place in France may have been affected by the epidemic and mitigation actions. This article presents mortality rate ratios by place of death in France during the first lockdown (17 March – 10 May, 2020) of the Covid-19 epidemic.

**Methods:**

We considered five places of death recorded in death certificates. Deaths in 2020 were compared to deaths from 2015 to 2019. We employed quasi-Poisson regressions in order to stablish mortality rate ratios (MRR) during the Covid-19 epidemic, for all-cause and non-Covid-19 deaths. Analysis was conducted in Metropolitan France, and for three groups of regions defined according to the intensity of the first COVID-19 epidemic wave.

**Results:**

A significant increase in all-cause and non-COVID-19 mortality at home was observed for all age groups. Also, an increase in mortality was observed in nursing homes, mostly due to Covid-19. Non-covid-19 mortality in public hospitals decreased significantly in all the country. These trends were mainly observed for cancers.

**Conclusions:**

Overall mortality increased during the first wave of the Covid-19 epidemic. Most Covid-19 deaths took place in public hospitals and nursing homes at old ages. There was a displacement of non-Covid-19 mortality from public hospitals to home and nursing homes, particularly in the most highly exposed area. Among hypotheses to explain such a displacement, population avoidance of hospital care, or redeployment of hospital activity in this emergent context can be cited. Further analysis is needed to understand the reasons of the increase in non-Covid-19 mortality in nursing homes and at home.

## Background

The Covid-19 epidemic has entailed – since its beginning in 2020 – a major health event in France, having caused more than 150,000 fatalities by mid-2022 [1]. Such an epidemic has challenged the efficiency and capacity of the public health system. During the first wave of the epidemic alone, lasting from March until May 2020, more than 25,000 excess deaths were observed in the country [2], in spite of the unprecedented sanitary measures that were implemented, particularly a strict lockdown. This excess was strongly higher in two regions and in a lesser extend in three other regions, while a limited or no excess was observed in the other regions.

As observed in other countries [3–5], those measures, alongside the capacity of public hospitals required by patients infected by Covid-19, might have distinctly affected the distribution of deaths by place during this first wave of the epidemic in France. Public hospitals are the most common place of death in France, accounting for almost half of the fatalities occurring in the country [6]. In addition, the distribution of deaths shows typically one fifth of deaths occurring in nursing homes, and another fifth at home. In spite of the potential disruption of these patterns particularly for non-Covid-19 deaths, no measure has yet been made of the magnitude of the impact of the Covid-19 epidemic on the distribution of deaths by place.

This paper aims to describe the changes in the distribution of deaths by place that have taken place in France during the first wave of the Covid-19 epidemic compared to the 2015–19 reference period. The potential changes were explored for both all-cause and non-Covid-19 mortality, and for three geographical zones that were differently impacted by the first wave of the Covid-19 epidemic.

## Methods

### Data source

All-cause and by cause individual mortality data come from death certificates collected and processed by CépiDc-Inserm, the French epidemiological centre for medical causes of death [7]. It is responsible for the French national causes of death database and it implements the coding process following WHO rules, from the 10th revision of the International Classification of Diseases (i.e., ICD10). These individual data include information on the age, sex, municipality of residence and death, date of death, place of death and cause of death for each individual. Only individuals having died within the French territory are included. We will consider here all-cause mortality data from 2015 until 2020, and mortality data by cause from 2014 to 2016, as data on deaths by cause for the period 2017–2019 has not been coded in ICD10 yet. Data by cause before 2014 were not considered due to distance with the study period. Data by cause for the period from March to May 2020 were also used, since Inserm-CépiDc chose to prioritize the coding of these data due to the emergence of the severe Covid-19 epidemic. Analysis by cause was based on the underlying cause of death.

Due to the slow delivery and processing of death certificates [7], all-cause CépiDc-Inserm mortality data is not complete for the period 2017–2019 (about 2% missing data). In order to correct this data, it has been compared to mortality data published by the National Institute of Statistics and Economic Studies (Insee) [8]. These Insee data provide the most up-to-date number of deaths by age, sex, date of death and department of death, and will be considered as a reference in this paper. Under the assumption that the missing deaths by place in CépiDc-Inserm database are similarly distributed that the other deaths, we have extrapolated the number of deaths by place of death, using the marginal distribution of deaths from Insee database by age, sex, date of death and department of death.

Additionally, population data have been obtained from Insee, referring to January 1^st^ of successive years, and have been then linearly interpolated in order to obtain population by age and sex, at a monthly frequency.

The use of mortality data in the frame of public health surveillance has been authorized by the French National Commission for Data protection and Liberties (CNIL).

### Statistical analysis

This study is a retrospective time-series analysis. The study period corresponds to the first lockdown implemented in France (March 17 – May 10, 2020). For the analyses on the previous years (2015 to 2019), we also used the March 17 – May 10 period, for comparison purposes.

For this study, we grouped the 13 metropolitan French regions in three different groups (“zones”), based on the magnitude of the impact of the first wave of the Covid-19 epidemic. This magnitude is based on the excess mortality measured in a previous study [2] (regions with an excess over 20%, between 15 and 20%, and below 15%). This categorization was consistent with hospitalization rate, incidence and mortality rate measured in other studies [9–11]. We therefore consider the following exposure zones: high-exposure zone (Île-de-France and Grand Est regions, henceforth “HE”), medium-exposure zone (Hauts-de-France, Auvergne-Rhône-Alpes and Bourgogne-Franche-Comté regions, henceforth “ME”) and low-exposure zone (rest of metropolitan France, henceforth “LE”).

We examined five main different places of death, as they are defined in French death certificates: public hospitals (accounting for 47% of deaths for the study period in 2015–2019), at home (21%), nursing homes (19%), private hospitals (9%) and public places (1%). Because of the low number of deaths for people aged below 70 years old reported in nursing homes, all-cause mortality was analysed for people aged 50 years old and older, and mortality by cause was analysed only for people aged 70 years old and older.

For the year 2020, we considered primarily all-cause deaths (113,559 in the study period) and deaths with Covid-19 as the underlying coded cause of death (ICD10 codes U07.1 and U07.2). We defined non-Covid-19 deaths as the deaths whose the underlying cause was not from Covid-19.

Mortality data were stratified by exposure zone, place of death and large age group (0–49, 50–69, and 70 years old and over). For each stratum, two quasi-Poisson regressions were performed in order to estimate two mortality rate ratios (MRR), that represent the variations in all-cause and non-Covid-19 mortality respectively during the first lockdown period of the Covid-19 epidemic. The use of quasi-Poisson regressions is compelled by the considerable overdispersion in mortality data, making variance much larger than the mean, breaking thus the premise of a Poisson regression. Each of these generalized linear models considers sex, age (in 10-years age groups) and the interaction between both. Additionally, the year (as a continuous variable) was included in the models, in order to separate trends within the 6-year period we consider from the particular effect of 2020. Population was also included in the model as an offset.

Therefore, if we consider j 10-year age groups {A1, …, Aj}, the model is defined by the following equation:

1 $$\mathrm{log}\left(D\right)=\mathrm{log}\left(Pop\right)+{\beta }_{0}+\sum_{i=1}^{J}{\beta }_{i}\times I\left(a=i\right)+{\beta }_{J+1}\times s+\sum_{i=J+2}^{2J+1}{\beta }_{i}\times I\left(a=i\right)\times s+{\beta }_{2J+2}\times Year+{\beta }_{2J+3} \times I\left(Year=2020\right)$$

where *D* and *Pop* are deaths and population counts;${\beta }_{1}$ to ${\beta }_{j}$ are the effects of $J$ 10-year age groups on $\mathrm{log}\left(D\right)$; $I\left(a={A}_{i}\right)$ is a dummy variable equal to 1 for successive age groups ${A}_{i}$; ${\beta }_{j+1}$ is the effect of sex on $\mathrm{log}\left(D\right)$; $s$ is a dummy variable equal to 1 for women; ${\beta }_{J+2}$ to ${\beta }_{2J+1}$ are the effects of sex-age interaction on $\mathrm{log}\left(D\right)$; ${\beta }_{2J+2}$ is the effect of the year on $\mathrm{log}\left(D\right)$; $Year$ is the year (continuous) between 2015 and 2020; ${\beta }_{2J+3}$ is the effect of the Covid-19 epidemic period on $\mathrm{log}\left(D\right)$; and $I(Year=2020)$ is a dummy variable equal to 1 when the year is 2020.

Therefore, for each stratum, the mortality rate ratio is:

2 $$MRR=\mathrm{exp}\left({\beta }_{2J+3}\right)$$

Each place of death is considered separately and it is attributed the population of the exposure zone under consideration. Therefore, MRRs must be interpreted as ratios between age and sex-standardized crude mortality rates. Additionally, it is important to note that non-Covid-19 MRRs must never be interpreted as the expected outcome without the Covid-19 epidemic, but rather as the MRR for all the death causes save Covid-19.

A stratification by sex of the model (1) was carried out, in order to show the sex disparities of the mortality dynamics that we analysed.

### Analysis by cause of death

Other than Covid-19, we analysed the two principal causes of death in France: cardiovascular diseases and cancers, as the underlying cause in the death certificate. There are two main reasons why we limit ourselves to those two. In the first place, as they are – by far – the two main causes of death, other causes might not have a sufficient number of deaths during the study period to be able to draw conclusions. Moreover, as we only have 2014 to 2016 and 2020 mortality data with cause, there might be some time-trends in less common causes of death that we would not be able to detect and that would distort our conclusions.

Thus we compared the 2020 mortality rate for each of both aforementioned categories of medical causes, place of death and age group to the mean of the 2014–2016 mortality rates for the period of the year and establish the variation.

## Results

Considering France in its whole, about half of the Covid-19 deaths registered for the study period took place in public hospitals (14,450 out of 28,017). Additionnaly, 8,827 deaths took place in nursing homes, 3,175 in private hospitals, 1,451 at home and 114 in other places. Moreover, 15,229 of the 28,017 Covid-19 deaths (54.3%) took place in the high-exposure zone (HE), while 7,271 in the ME and 5,507 in the LE.

**Fig. 1:**
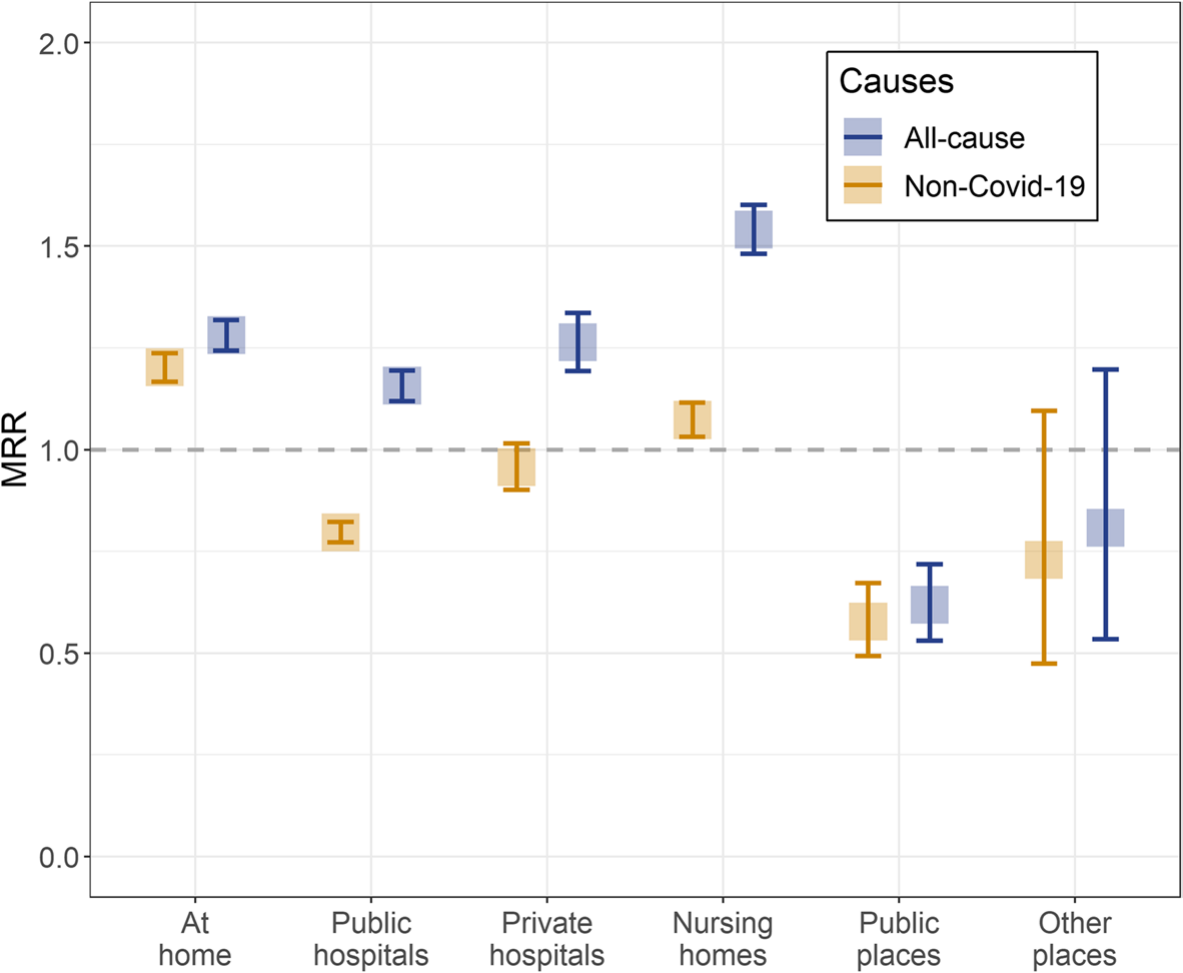
Age and sex-standardized mortality rate ratios by place of death, all-cause and non-Covid-19, from 17 March-10 May, 2020, compared with the same period from 2015 to 2019, Metropolitan France

During the first lockdown period (17 March-10 May, 2020), MRRs indicate significant increases in all-cause mortality in nursing homes (1.54 [IC95%, 1.48–1.60]), at home and in private and public hospitals (Fig. 1), while there was a significant decrease in all-cause mortality in public places (MRR = 0.62 [IC95%, 0.53–0.72]).A significant decrease in non-Covid-19 mortality was observed in public hospitals (MRR = 0.80 [IC95%, 0.77–0.82]) and non significantly in private hospitals, while MRR remained significantly higher than 1 in nursing homes (1.07 [IC95%, 1.03–1.12]) and, stronger, at home (1.20 [IC95%, 1.17–1.24]) (Fig. 1).

**Fig. 2:**
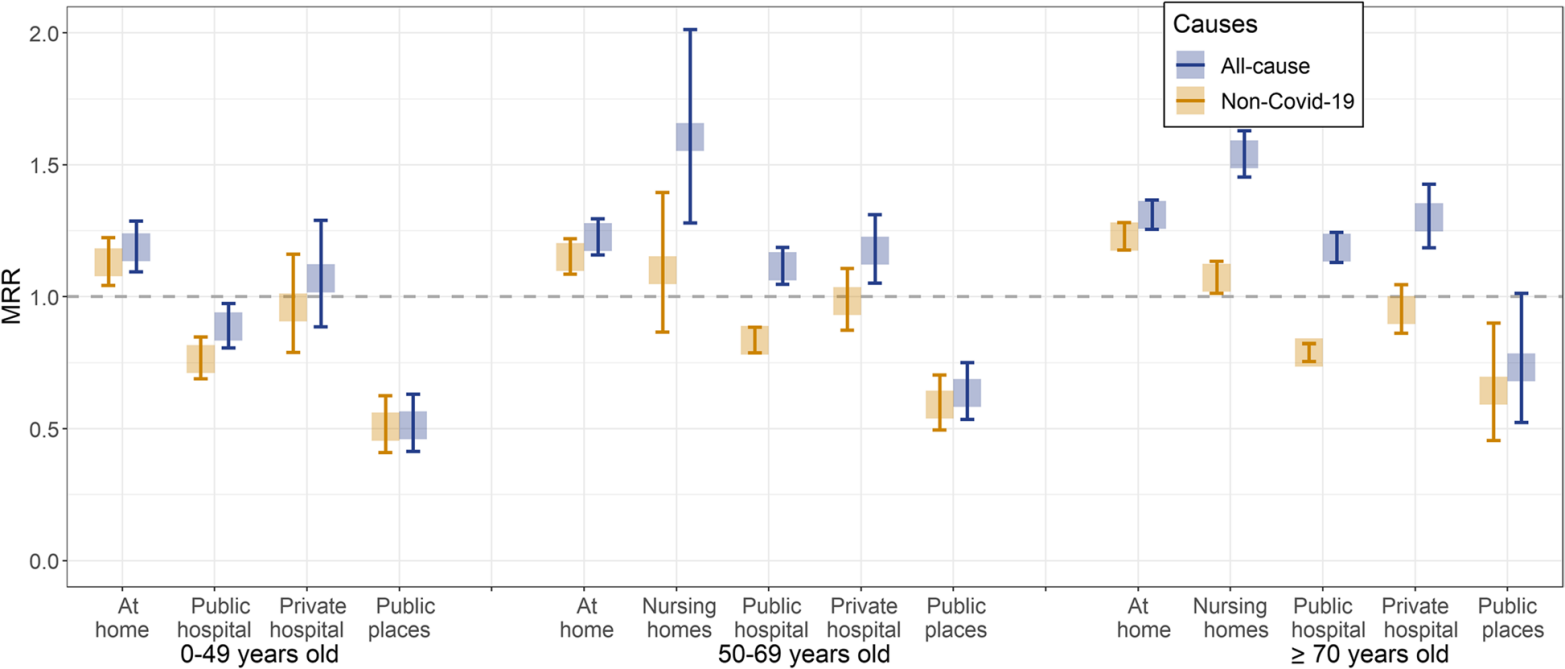
Age and sex-standardized mortality rate ratios by place of death and age groups, from 17 March-10 May, 2020, compared with the same period from 2015 to 2019, Metropolitan France

Covid-19 deaths accounted for the most part of mortality increase in nursing homes and private and public hospitals, while they only explained a very limited part of the mortality increase observed at home. Results were similar for each sex but varied strongly with age (Fig. 2). Considering all causes of deaths, a significant increase was observed in public and private hospitals for the population aged 50 years old and over, while it decreased significantly for the population under 50 years old in public hospitals (MRR = 0.89 [IC95%, 0.81–0.97]) (Fig. 2). Further analysis carried out by sex show that this decrease took place only for men. At home, a significant increase was observed for all three age groups, higher for the elderly (1.31 [IC95%, 1.26–1.31]) (Fig. 2). In nursing homes, we also observed a significant increase for people aged 50 years old and over.

Considering non-Covid-19 deaths, the observed decrease in mortality in public hospitals seemed similar for all three age groups. Moreover, the MRRs at home remained significantly over 1 and were similar for the three age groups (Fig. 2).

While all-cause MRR and non-Covid-19 MRR were close for people aged 0–49 years old, the results suggested that differences in the all-cause and non-Covid-19 MMRs increased with age (except at home and in public places) since Covid-19 deaths were more numerous for the elderly (Fig. 2).

MRRs for all-cause and non-Covid-19 mortality in the different places of death varied according to the magnitude of the epidemic (Table 1). For the population aged under 50 years old, a significant increase in all-cause (1.46 [IC95%, 1.20–1.77]) and non-Covid-19 (1.31 [IC95%, 1.07–1.60]) mortality at home was observed only in the high-exposure zone (HE). In addition, in public hospitals, we observed a significant decrease of all-cause mortality only in the medium and low-exposure zones (ME and LE), while non-covid mortality significantly decreased in all three zones.

**Table 1:** Age and sex-standardized mortality rate ratios by place of death, age group and exposure zone, from 17 March-10 May, 2020, compared with the same period in 2015–2019, Metropolitan France

		**00–49 years old**				**50–69 years old**				**70 years old and over**			
		**All-cause**		**Non-Covid-19**		**All-cause**		**Non-Covid-19**		**All-cause**		**Non-Covid-19**	
	**Zone**^a^	**MRR**^b^	**CI95%**	**MRR**^b^	**CI95%**	**MRR**^b^	**CI95%**	**MRR**^b^	**CI95%**	**MRR**^b^	**CI95%**	**MRR**^b^	**CI95%**
**At home**	HE	1.46	[1.20–1.77]	1.31	[1.07–1.60]	1.43	[1.29–1.58]	1.23	[1.10–1.37]	1.62	[1.51–1.73]	1.40	[1.31–1.49]
	ME	1.17	[1.01–1.36]	1.14	[0.98–1.32]	1.24	[1.12–1.37]	1.17	[1.06–1.30]	1.28	[1.21–1.36]	1.20	[1.13–1.27]
	LE	1.09	[0.98–1.22]	1.06	[0.95–1.18]	1.15	[1.07–1.22]	1.11	[1.04–1.19]	1.22	[1.16–1.29]	1.19	[1.13–1.25]
**Nursing homes**	HE					3.69	[2.85–4.80]	1.61	[1.18–2.21]	2.89	[2.64–3.17]	1.38	[1.28–1.48]
	ME					1.21	[0.88–1.67]	0.85	[0.62–1.16]	1.50	[1.38–1.62]	1.00	[0.92–1.08]
	LE					1.22	[0.85–1.74]	1.10	[0.76–1.58]	1.16	[1.10–1.23]	1.02	[0.96–1.07]
**Private hospitals**	HE	1.08	[0.84–1.37]	0.87	[0.68–1.11]	1.36	[1.11–1.66]	0.89	[0.71–1.10]	1.76	[1.49–2.07]	0.96	[0.81–1.14]
	ME	0.96	[0.62–1.50]	0.88	[0.56–1.37]	1.07	[0.92–1.24]	0.96	[0.82–1.11]	1.27	[1.14–1.42]	0.92	[0.82–1.04]
	LE	1.04	[0.83–1.30]	1.01	[0.81–1.27]	1.07	[0.95–1.19]	1.02	[0.91–1.15]	1.04	[0.96–1.13]	0.94	[0.86–1.03]
**Public hospitals**	HE	1.09	[0.92–1.30]	0.80	[0.67–0.95]	1.65	[1.46–1.87]	0.89	[0.80–0.99]	1.68	[1.57–1.80]	0.76	[0.73–0.79]
	ME	0.83	[0.72–0.96]	0.77	[0.66–0.89]	1.03	[0.96–1.11]	0.81	[0.75–0.87]	1.18	[1.11–1.25]	0.78	[0.73–0.83]
	LE	0.79	[0.69–0.90]	0.74	[0.65–0.85]	0.91	[0.86–0.98]	0.82	[0.77–0.88]	0.98	[0.94–1.03]	0.81	[0.77–0.85]
**Public places**	HE	0.46	[0.33–0.64]	0.46	[0.33–0.64]	0.80	[0.41–1.51]	0.64	[0.31–1.24]	0.81	[0.50–1.29]	0.54	[0.32–0.91]
	ME	0.60	[0.42–0.84]	0.60	[0.42–0.84]	0.61	[0.46–0.81]	0.58	[0.44–0.78]	0.62	[0.37–1.02]	0.54	[0.31–0.90]
	LE	0.49	[0.37–0.64]	0.48	[0.36–0.63]	0.59	[0.49–0.72]	0.58	[0.47–0.70]	0.76	[0.52–1.10]	0.74	[0.50–1.07]

Mortality rate ratios from the quasi-Poisson model described by Eqs. (1) and (2) in the Methods section. Data have been stratified by large age group, place of death, and exposure zone

^a^*HE* High–exposure zone, *ME* Medium-exposure zone, *LE* Low-exposure zone

^b^*MRR* Mortality rate ratio, *CI95* 95% confidence interval

Considering the two other age groups, MRRs for all-cause and non-covid mortality at home were significantly over 1 in all exposure zones, with higher estimations in the HE (Table 1). In nursing homes, MRRs for both all-cause and non-covid mortality were significantly over 1 in the high-exposure zone (HE). MRRs for non-covid mortality reached 1.61 [CI95%, 1.18–2.21] for people aged 50–69 years old and 1.38 [CI95%, 1.28–1.48] for people aged 70 years and over. In the two other exposure zones (ME and LE), only MRRs of all-cause mortality were significantly over 1 for people aged 70 years old and over.

In public hospitals, non-Covid mortality significantly decreased in all exposure zones with comparable MRR, while all-cause mortality remained significantly over 1 in HE for the two older age groups and in the ME for the elderly (Table 1).

Finally, mortality rates during the first lockdown in 2020 decreased in public hospitals in comparison with the 2014–2016 reference period for cancers in all age groups, and – inversely – mortality rates for cancers increased at home (Table 2). Mortality rates for cardiovascular diseases at home also increased for people aged lesser than 70 years old in the high-exposure zone and to a lesser extent in the medium-exposure zone, but decreased in the low-exposure zone. In addition, particularly for those aged 70 years old and over, these trends of mortality rates for cancers and cardiovascular diseases in public hospitals, at home and in nursing homes seemed more important in the high-exposure zone.

﻿For the elderly, mortality rates for cancers and cardiovascular diseases in nursing homes increased in 2020 compared with the reference period in the high-exposure zone (+ 11% and + 14% respectively), while they slightly decreased in the two other zones.

**Table 2:** Crude mortality rates per 100,000 inhabitants for cancers and cardiovascular diseases by age group and place of death, Metropolitan France

Mortality rates (per 100,000)		Place of death	High-exposure zone			Medium-exposure zone			Low-exposure zone		
			2014–16 ^a^	2020 ^b^	Variation ^c^	2014–16 ^a^	2020 ^b^	Variation ^c^	2014–16 ^a^	2020﻿ ^b^	Variation ^c^
**0–49 years old**	Cancers	Public hospitals	1.8	1.2	-31%	1.8	1.2	-35%	1.8	1.2	-32%
		Home	0.3	0.5	55%	0.5	0.6	22%	0.4	0.5	32%
		**Total** ^d^	**2.5**	**2.4**	**-2%**	**2.7**	**2.1**	**-23%**	**2.7**	**2.3**	**-14%**
	Cardiovascular diseases	Public hospitals	0.5	0.3	-34%	0.5	0.4	-30%	0.4	0.3	-30%
		Home	0.3	0.3	36%	0.4	0.4	13%	0.4	0.3	-25%
		**Total** ^d^	**0.9**	**0.8**	**-14%**	**1.0**	**0.9**	**-15%**	**1.0**	**0.7**	**-26%**
**50–69 years old**	Cancers	Public hospitals	34.0	18.5	-45%	34.0	22.5	-34%	27.8	19.5	-30%
		Home	5.2	7.7	47%	9.0	12.2	35%	7.4	10.4	41%
		**Total** ^d^	**47.8**	**38.2**	**-20%**	**53.3**	**44.0**	**-17%**	**46.8**	**41.6**	**-11%**
	Cardiovascular diseases	Public hospitals	6.8	5.2	-24%	7.7	5.8	-25%	6.5	5.1	-21%
		Home	4.5	4.8	7%	6.1	6.1	1%	5.2	4.7	-9%
		**Total** ^d^	**13.5**	**12.4**	**-8%**	**16.1**	**13.7**	**-15%**	**13.8**	**11.4**	**-18%**
**70 years old or over**	Cancers	Public hospitals	116.7	63.2	-46%	104.6	74.4	-29%	85.4	61.4	-28%
		Nursing homes	16.6	18.4	11%	23.4	22.4	-4%	21.4	21.1	-1%
		Home	22.4	36.5	63%	31.2	41.9	34%	30.5	41.1	35%
		**Total** ^d^	**186.4**	**160.3**	**-14%**	**189.3**	**166.7**	**-12%**	**171.0**	**153.8**	**-10%**
	Cardiovascular diseases	Public hospitals	103.5	50.8	-51%	107.7	66.2	-39%	94.0	59.5	-37%
		Nursing homes	38.7	44.3	14%	52.4	48.6	-7%	51.9	49.0	-6%
		Home	40.7	40.6	0%	47.5	43.9	-8%	49.0	43.5	-11%
		**Total** ^d^	**202.5**	**155.1**	**-23%**	**225.6**	**172.6**	**-23%**	**215.7**	**166.7**	**-23%**

^a^Mean mortality rate of the 2014–2016 reference period (17 March – 10 May)

^b^Mortality rate on 2020 period (17 March – 10 May)

^c^Variation in percentage between both previous columns

^d^Total refers to the sum of all places of death (and not only those that are shown in upper rows)

## Discussion

The results showed an increase of all-cause mortality during the first lockdown from March 17 to May 10, 2020 in all places of death (except public places) for people older than 50 years old in metropolitan France, as well as for people aged 0–49 years old at home. The biggest impact is observed in nursing homes for people aged 50 years old and over in the high-exposure zone. In public places, mortality decreased for all age groups and all exposure zones.

The results also showed a possible shift of non-Covid-19 deaths from public hospitals to home and nursing homes during the first lockdown, particularly for people aged 50 years old and over and in the high-exposure zone. At home, non-Covid-19 mortality remained significantly in excess for people aged 0–49 years old in the high-exposure zone, and in all geographical zones for people aged 50 years old and over. In nursing homes, for people older than 50 years old, a significant increase of non-Covid-19 mortality was also observed in the high-exposure zone. Inversely, in public hospitals, non-Covid-19 mortality decreased significantly for all age groups and in all exposure zones.

Among non-Covid-19 mortality, the shift was observed for cancers in all exposure zones and to a lesser extent, for cardiovascular diseases in the high-exposure zone.

While studies have explored the evolution of in-hospital morbidity and mortality during the Covid-19 epidemic [12–14], few studies have analysed the distribution of mortality according to the place of death. The trends by place of death for all-cause and non-Covid-19 mortality observed in our study are consistent with those observed in England and Wales [3, 4], the United-States [15] and Denmark concerning deaths by cardiovascular diseases [5]. In particular, a prominent all-cause mortality increase in nursing homes was also shown in England and Wales [16]. These results confirmed that only a limited part of mortality observed at home was attributable to Covid-19, while Covid-19 mortality mainly occurred in hospitals and in nursing homes. We cannot exclude that Covid-19 deaths may be slightly underreported, since symptoms were not specific and in absence of laboratory tests.

Our results on non-Covid-19 mortality suggest a displacement of a part of mortality for other causes during the first wave of the Covid-19 epidemic from public hospitals to nursing homes and, more generally, home for all age groups and in all three zones. Several hypotheses can be put forward to explain this displacement. First, it may be reflecting a population avoidance of hospital care during the lockdown period, by fear of contamination or to prevent hospitals being overwhelmed. This was also observed in the admission trends in hospitals and in emergency departments [17, 18]. This behaviour may have conducted to a delay in diagnosis of diseases, increasing risk of death.

Further, at the beginning of the spread of the epidemic among the population, a redeployment of hospital activity was done in order to concentrate their healthcare capacity on managing urgent diseases, and in prevision of a massive influx of Covid-19 patients. The hospital-at-home system was also used more frequently in 2020 for patients in palliative situations and in chemotherapy [19].

In the context of this emergent disease and in absence of Covid-19 testing at the beginning of the epidemic, we could also guess that patients in nursing homes were transferred less than usually to hospital, in order to limit the spread of the epidemic, since nursing homes were strongly affected by the epidemic. Transfers could also be limited to avoid the hospitals being overwhelmed, particularly in the case of patients in palliative situations.

Our results on mortality rates for cancers and for cardiovascular diseases have to be interpreted cautiously, since the observed variations may be partly due to the usual trend of mortality between 2020 and the reference period (2014–2016). However, our results are consistent with the other international studies [5, 20]. A reduction of admissions in hospitals and in emergency departments for cardiovascular diseases (stroke, myocardial infarction) [21–23] or for cancers [24] during the lockdown was observed in France, as well as in other countries [22, 25]. Beyond the hypotheses described above, this decrease could be a positive consequence of the activity decline that the population experienced during the lockdown (less daily stress, reduction of air pollution [26, 27], …).

Moreover, excepting comorbidities, individual information on the context and healthcare of the patient prior to death are too limited in death certificates to allow us to analyse characteristics of patients who died at home or in nursing homes during the lockdown. Complementary studies using data from the National Hospitalization Database (PMSI) and about the reimbursements of medical care could be conducted in order to better understand the health status of non-Covid-19 patients dying during the lockdown at home and in nursing homes, compared to the reference period.

## Limitations

Since the results were obtained by stratifying the statistical model by age group and by place of death, MRR by age group cannot be directly compared to each other. A more sophisticated statistical model, including age group and place of deaths, would be necessary to verify if the differences in the all-cause and non-Covid-19 MRR significantly increased with age in public and private hospitals and in nursing homes, as suggested by our results. This advanced model could also improve the estimate of a potential change of the trend of mortality in 2020. In addition, further analysis needs to be conducted and extended to all categories of medical causes of deaths, using robust statistical analysis including long-term trends and mortality data by cause during the 2017–2019 period, among other parameters. Such analysis will enable to identify whether the displacement of mortality from hospitals to home and nursing homes concerned all causes of deaths.

Finally, this study was restricted to the first lockdown period. When medical causes of deaths for the rest of 2020 and 2021 are coded and available, it would be interesting to explore the distribution of mortality by place of death after and during the two other lockdowns (in November 2020 and April 2021, respectively). Comparing mortality of different phases of the epidemic would allow us to know more about the effectiveness of the lockdown.

## Conclusion

Beyond specific systems implemented at the beginning of the epidemic to count Covid-19 cases and deaths in hospitals and nursing homes in France, mortality surveillance during the epidemic was also based on civil-status offices (all-cause mortality) and Electronic Death Registration System (mortality by medical cause). However, this last system is not deployed uniformly according to region and place of death: it over-represents hospital-based deaths but collects less than 5% of deaths at home and 15% of deaths in nursing homes. Wider deployment of electronic death certification, particularly in nursing homes and in private homes, is required to ensure reactive surveillance of mortality by cause in all places, since other impact of such major epidemics, or the impact of other mortality events (such as heatwaves in the summer period) may occur simultaneously.

## Data Availability

The complete dataset of this research is only available under strictly secured conditions as it contains individual data protected by French legislation. Aggregated data could be shared on reasonable request to the corresponding author, Anne Fouillet (anne.fouillet@santepubliquefrance.fr).

## Abbreviations

CNIL: French National Commission for Data protection and Liberties
HE: High exposure zone
ICD10: International classification of diseases 10th revision
Insee: French National Institute of Statistics and Economic Studies
LE: Low exposure zone
ME: Medium exposure zone
MRR: Mortality rate ratios
